# Особенности стероидогенеза и артериальная гипертензия у мужчин при разных типах «физиологической» гиперандрогении у мужчин

**DOI:** 10.14341/probl13226

**Published:** 2023-05-11

**Authors:** В. А. Филатова, Р. В. Роживанов, И. З. Бондаренко, В. А. Иоутси, Е. Н. Андреева, Г. А. Мельниченко, Н. Г. Мокрышева

**Affiliations:** Национальный медицинский исследовательский центр эндокринологии; Национальный медицинский исследовательский центр эндокринологии; Национальный медицинский исследовательский центр эндокринологии; Национальный медицинский исследовательский центр эндокринологии; Национальный медицинский исследовательский центр эндокринологии; Национальный медицинский исследовательский центр эндокринологии; Национальный медицинский исследовательский центр эндокринологии

**Keywords:** гиперандрогения, тестостерон, дигидротестостерон, мужчины, артериальная гипертензия

## Abstract

**ЦЕЛЬ:**

ЦЕЛЬ. Выявить особенности стероидогенеза и артериальной гипертензии при «физиологической» гиперандрогении у мужчин.

**МАТЕРИАЛЫ И МЕТОДЫ:**

МАТЕРИАЛЫ И МЕТОДЫ. Сплошное одномоментное исследование. Сравнивались группы мужчин с гиперандрогенией, обусловленной повышением уровня общего тестостерона (n=34), и гиперандрогенией, обусловленной повышением уровня дигидротестостерона (ДГТ) (n=66). При определении типа гиперандрогении и распределении пациентов по группам уровни ДГТ и общего тестостерона определялись методом усиленной хемилюминесценции. В группе пациентов с гиперандрогенией, обусловленной повышением общего тестостерона, проводилось сравнение подгрупп мужчин с артериальной гипертензией и без таковой. У всех пациентов оценивались индекс массы тела, величина окружности талии, величины систолического и диастолического артериального давления, оценивался пульс, определялись показатели уровней лютеинизирующего гормона, глобулина, связывающего половые стероиды, эстрадиола, мультистероидного анализа крови (методом тандемной масс-спектрометрии), глюкозы, липидного спектра крови, мочевой кислоты, креатинина, ренина, калия, натрия, хлорида крови. Пациентам с артериальной гипертензией дополнительно проводились суточное мониторирование АД, оценка альбуминурии, электрокардиография, осмотр глазного дна. Базовый пороговый уровень значимости p<0,05. При множественных сравнениях проводился расчет уровня значимости p с применением поправки Бонферрони.

**РЕЗУЛЬТАТЫ:**

РЕЗУЛЬТАТЫ. Были выявлены статистически значимые различия в уровнях 17-гидроксипрегненолона, 17-гидрокси-прогестерона и андростендиона, которые были выше у мужчин с повышенным уровнем общего тестостерона. Статистически значимых различий в других лабораторных параметрах выявлено не было. Случаев повышения артериального давления в группе мужчин с повышением ДГТ выявлено не было. В группе мужчин с повышением уровня общего тестостерона было выявлено 23,5% мужчин с артериальной гипертензией без поражения органов-мишеней, с гиперандрогенией было ассоциировано 17,6% случаев. Для артериальной гипертензии, ассоциированной с гиперандрогенией, был характерен подъем артериального давления в ранние утренние часы. Уровни эстрадиола, оставаясь в пределах нормы, были статистически значимо ниже у пациентов с артериальной гипертензией в сравнении с мужчинами с повышенным тестостероном, но без гипертензии.

**ЗАКЛЮЧЕНИЕ:**

ЗАКЛЮЧЕНИЕ. При «физиологической» гиперандрогении, обусловленной повышением уровня ДГТ, случаев артериальной гипертензии не наблюдается, в то время как частота ее встречаемости при «физиологической» гиперандрогении, обусловленной повышением общего тестостерона, составила 23,5%. Особенностями стероидогенеза являлись повышенная выработка 17-гидроксипрегненолона, 17-гидроксипрогестерона и андростендиона у мужчин с тестостероновой гиперандрогенией и сниженная выработка эстрадиола у пациентов с артериальной гипертензией в сравнении с пациентами без таковой при тестостероновой гиперандрогении.

## Abstract (en)

**AIM:**

AIM. To reveal the peculiarities of steroidogenesis and arterial hypertension in «physiological» hyperandrogenism in men.

**MATERIALS AND METHODS:**

MATERIALS AND METHODS. One-stage simultaneous study. The groups of men with hyperandrogenism caused by increased total testosterone (n=34) and those with hyperandrogenism caused by increased dihydrotestosterone (DHT) (n=66) were compared. In determining the type of hyperandrogenism and allocating patients to groups, DHT and total testosterone levels were determined by enhanced chemiluminescence. Subgroups of men with and without arterial hypertension were compared in the group of patients with hyperandrogenism due to an increase in total testosterone. Body mass index, waist circumference, systolic and diastolic blood pressure, pulse, and LH, SBHG, estradiol, blood multisteroid levels by isotope dilution liquid chromatography/tandem mass spectrometry, glucose, blood lipid spectrum, uric acid, creatinine, renin, potassium, sodium, and blood chloride were assessed in all patients. Patients with arterial hypertension additionally underwent daily BP monitoring, albuminuria assessment, electrocardiography, ocular fundus examination. The baseline threshold level of significance was p<0.05. For multiple comparisons, the p significance level was calculated using the Bonferroni correction.

**RESULTS:**

RESULTS. Statistically significant differences were found in the levels of 17-hydroxypregnenolone, 17-hydroxyprogesterone, and androstenedione, which were higher in men with elevated levels of total testosterone. No statistically significant differences in other laboratory parameters were found. No cases of increased blood pressure were detected in the group of men with elevated DHT. In the group of men with elevated total testosterone, 23,5% of men with arterial hypertension without targetorgan lesions were identified, while hyperandrogenism was associated with 17,6% of cases. Arterial hypertension associated with hyperandrogenism was characterized by a rise in blood pressure in the early morning hours. Estradiol levels, while remaining within normal limits, were statistically significantly lower in patients with arterial hypertension compared with men with elevated testosterone but without hypertension.

**CONCLUSION:**

CONCLUSION. No cases of arterial hypertension were observed in «physiological» hyperandrogenism due to elevated DHT levels, whereas its incidence in «physiological» hyperandrogenism due to elevated total testosterone was 23,5%. The features of steroidogenesis were increased production of 17-hydroxypregnenolone, 17-hydroxyprogesterone, and androstenedione in men with testosterone hyperandrogenism and decreased estradiol production in patients with arterial hypertension compared with patients without testosterone hyperandrogenism.

## ОБОСНОВАНИЕ

Термин «гиперандрогения» подразумевает избыточный синтез андрогенных гормонов [1]. В настоящее время проведено множество исследований и представлены данные по распространению гиперандрогении у женщин с такими заболеваниями, как синдром поликистозных яичников (СПКЯ) или врожденная дисфункция коры надпочечников (ВДКН). Большая часть исследований гиперандрогении у мужчин посвящена вопросам андрогенной алопеции, в которых, тем не менее, не рассматривается системное влияние андрогенов на метаболические процессы и артериальное давление (АД) [2][3]. Гиперандрогения у мужчин встречается, как правило, в молодом возрасте, при этом, по данным проведенного метаанализа, молодые люди с повышенным АД имеют повышенный риск сердечно-сосудистых заболеваний в более позднем возрасте [4]. По данным исследования Gillis E.E. и соавт. (2016), мужчины более склонны к гипертонии, чем женщины, что отмечается на протяжении большей части жизни и особенно в молодом возрасте; эта разница между полами с возрастом уменьшается и при сравнении старших возрастных групп с женщинами после менопаузы становится несущественной [5]. Также известно, что повышение АД часто отмечается у мужчин, имеющих повышенный уровень тестостерона, за счет использования андрогенных анаболических стероидов [6]. В исследовании Chasland L.C. и соавт. (2021) было выявлено, что пациенты с регулярной физической нагрузкой, принимающие заместительную терапию препаратами тестостерона, имели более высокие показатели суточного АД по сравнению группой, принимавшей плацебо [7]. В связи с этим вопрос изучения ассоциации артериальной гипертензии у мужчин c физиологической гиперандрогенией (обусловленной гиперпродукцией тестостерона и/или дигидротестостерона (ДГТ) при нормальных уровнях лютеинизирующего гормона (ЛГ) и глобулина, связывающего половые гормоны (ГСПГ) в отсутствие ВДКН) требует изучения [8].

## ЦЕЛЬ ИССЛЕДОВАНИЯ

Выявить особенности стероидогенеза и артериальной гипертензии при «физиологической» гиперандрогении у мужчин.

## МАТЕРИАЛЫ И МЕТОДЫ

Место и время проведения исследования

ФГБУ «НМИЦ эндокринологии» Министерства здравоохранения РФ, Москва. Исследование выполнено в период с сентября 2020 по декабрь 2022 г.

Изучаемые популяции

Формирование групп проводилось из пациентов, обратившихся за медицинской помощью в ФГБУ «НМИЦ эндокринологии» Министерства здравоохранения РФ.

Критерии включения: повышенный уровень ДГТ и/или общего тестостерона (основной критерий включения), мужской пол.

Критерии невключения: повышенный или сниженный уровень ЛГ, повышенный уровень ГСПГ, индекс массы тела (ИМТ) >30 кг/м2, патологические изменения любого из яичек при осмотре, нарушения кариотипа, задержка полового развития, наличие в анамнезе крипторхизма, воспалительных заболеваний, опухолей, травм или хирургических вмешательств на половых органах и области головного мозга, включая гипофиз, средняя или тяжелая перенесенная инфекция COVID-19, сахарный диабет, гипотиреоз, тиреотоксикоз, любые заболевания надпочечников, курение, употребление алкоголя чаще 1 раза в 4 нед, постоянный прием любых медицинских препаратов, врожденные заболевания сердечно-сосудистой системы.

Критерии исключения: отказ от выполнения программы исследования (все проводимые исследования являлись рутинными, однако у пациентов было право отказаться от них полностью или частично, и такие пациенты в исследование не включались).

Способ формирования выборки из изучаемой популяции

Выборка формировалась сплошным способом.

Дизайн исследования

Сплошное одномоментное исследование мужчин с повышенным уровнем общего тестостерона и/или ДГТ. На основании оценки уровня ДГТ и общего тестостерона были выявлены пациенты с гиперандрогенией, обусловленной изолированным повышением общего тестостерона (n=29), а также повышением уровня общего тестостерона в сочетании с повышенным уровнем ДГТ (n=5), которые были объединены в одну исследовательскую группу из-за малочисленности. Другую исследовательскую группу составили пациенты с гиперандрогенией, обусловленной изолированным повышением ДГТ (n=66). Кроме того, в группе пациентов с «физиологической» гиперандрогенией, обусловленной повышением общего тестостерона, проводилось сравнение подгрупп мужчин с артериальной гипертензией и без таковой.

Методы

Физикальное обследование включало общий осмотр с определением характеристик оволосения, в том числе лобкового, типа телосложения, состояния грудных желез, а также оценку состояния и степени развития наружных половых органов. У всех пациентов регистрировались следующие результаты обследования: ИМТ, величина окружности талии (ОТ), величины систолического артериального давления (САД) и диастолического артериального давления (ДАД), частота сердечных сокращений, оценивался пульс с целью исключения нарушений ритма, определялись показатели уровней ДГТ, общего тестостерона, ЛГ, ГСПГ, эстрадиола, мультистероидного анализа крови, глюкозы, липидного спектра крови, мочевой кислоты, креатинина (с дальнейшим рассчетом СКФ по формуле CKD-EPI), ренина, калия, натрия, хлорида крови.

Поскольку в нашем исследовании не было пациентов с нарушением ритма, применялись автоматические устройства для измерения АД. Пациенты записывали результаты измерений АД в дневник самоконтроля 4 раза в сутки, при измерении сразу после сна; днем; вечером и на ночь. При статистической обработке результатов использовалось среднее арифметическое значение АД для каждого времени дня за 14 суток наблюдений. При установке категории АД использовали средние значения САД и ДАД в соответствии с клиническими рекомендациями [9]. Пациентам с артериальной гипертензией (синдром повышения САД >140 мм рт. ст. и/или ДАД >90 мм рт. ст.) дополнительно проводились: суточное мониторирование АД (СМАД), оценка альбуминурии (на лабораторном анализаторе «DCA 2000+» производства фирмы «Bayer» (Германия) методом ингибирования реакции латекс-агглютинации, тест считался положительным, если концентрация альбумина в моче превышала 20 мг/л в разовой порции мочи), электрокардиография (ЭКГ) в покое лежа в 12 стандартных отведениях, осмотр глазного дна методами обратной и прямой офтальмоскопии офтальмоскопами фирмы «Keeler» и «Sckepens-Pomeranceff» MIRA последовательно от центра до крайней периферии, во всех меридианах, с осмотром диска зрительного нерва и макулярной области. У пациентов с артериальной гипертензией оценивались факторы риска, сердечно-сосудистый риск и стадия заболевания в соответствии с клиническими рекомендациями [9].

При определении типа гиперандрогении и распределении пациентов по исследовательским группам уровни ДГТ (референсный интервал (РИ) 220–900 пг/мл) и общего тестостерона (РИ 12,0–33,0 нмоль/л) определялись на автоматическом анализаторе Vitros 3600 (Johnson and Johnson, США) методом усиленной хемилюминесценции. Уровни ЛГ (РИ 2,5–11,0 ЕД/л), эстрадиола (РИ 19,7–242 пмоль/л) и ГСПГ (РИ 20,6–76,7 нмоль/л) также определялись на автоматическом анализаторе Vitros 3600 (Johnson and Johnson, США) тем же методом. При оценке стероидогенеза определение общего тестостерона (РИ 12,0–33,0 нмоль/л), кортизола (РИ 140–630 нмоль/л), кортизона (РИ 33–97 нмоль/л), 21-деоксикортизола (РИ 0–1,2 нмоль/л), 11-дезоксикортизола (РИ 0–10 нмоль/л), альдостерона (РИ 70–980 пмоль/л, в положении сидя), кортикостерона (РИ 1–50 нмоль/л), деоксикортикостерона (0–0,58 нмоль/л), прегненолона (РИ 0–7 нмоль/л), прогестерона (РИ 0,1–1,0 нмоль/л), 17-гидроксипрегненолона (РИ 0–20 нмоль/л), 17-гидроксипрогестерона (РИ 0,2–6,0 нмоль/л), дегидроэпиандростерона (РИ 4–50 нмоль/л), андростендиона (РИ 0,8–9,0 нмоль/л) выполнялось с помощью метода высокоэффективной жидкостной хроматографии с тандемной масс-спектрометрией (ВЭЖХ-МС/МС) на хроматографе Agilint 1290 Infinity II, масс-спектрометре AB Sciex TripleQuad 5500. Концентрацию биохимических показателей сыворотки крови — холестерина (ХС) (РИ 3,3–5,2 ммоль/л), холестерина липопротеидов низкой плотности (ЛПНП) (РИ 1,1–3,0 ммоль/л), холестерина липопротеидов высокой плотности (ЛПВП) (РИ 0,9–2,6 ммоль/л), триглицеридов (ТГ) (РИ 0,1–1,7 ммоль/л), глюкозы (РИ 3,1–6,1 ммоль/л), мочевой кислоты (РИ 202–416 мкмоль/л), креатинина (РИ 63–110 мкмоль/л), ренина (РИ 2,8–39,9 мЕд/л), калия (РИ 3,5–5,1 ммоль/л), натрия (РИ 136–145 ммоль/л), хлорида (РИ 98–107 ммоль/л) определяли на биохимическом анализаторе Hitachi (Biohringer Mannheim, Япония).

Описание медицинского вмешательства

Всем пациентам осуществлялся забор крови в пробирки типа «вакутейнер» в утреннее время натощак из локтевой вены.

Всем мужчинам с артериальной гипертензией были даны рекомендации по изменению образа жизни на 3 мес: целевым давлением являлось АД менее 130/80 мм рт. ст., но не менее 120/70 мм рт. ст., достижение целевого АД — 3 мес. Был рекомендован ночной сон не менее 7 ч в сутки, нормализация массы тела для лиц с ее избытком. Рекомендовано ограничение потребления соли до 5 г/сут, увеличение потребления калия. При гиподинамии (сидячая работа более 5 ч/сут, физическая активность менее 10 ч/нед) рекомендовано применять регулярные физические тренировки не менее 4 раз в неделю продолжительностью 30–45 минут. При физической нагрузке число сердечных сокращений должно увеличиваться не более чем на 20–30 в 1 мин. Рекомендовано ограничить продолжительность рабочего дня и домашних нагрузок, избегать ночных смен, командировок.

Статистический анализ

Принципы расчета размера выборки

Размер выборки предварительно не рассчитывался. Включали всех обратившихся пациентов, соответствующих критериям исследования.

Методы статистического анализа данных

Статистическая обработка полученных данных была проведена с использованием пакета прикладных программ STATISTICA (Stat Soft Inc., США, версия 8.0) [10]. Поскольку объемы выборок были небольшими, а распределения признаков не являлись нормальными, сравнение по количественным признакам осуществлялось непараметрическим методом с использованием U-критерия Манна–Уитни, а по качественным — двустороннего точного критерия Фишера. Базовый пороговый уровень значимости p<0,05. При множественных сравнениях проводился перерасчет уровня значимости p с применением поправки Бонферрони. Результаты исследований представлены в виде медиан и границ интерквартильного отрезка для количественных признаков, а также абсолютных чисел и процентов для качественных признаков.

Этическая экспертиза

Проведение исследования одобрено Локальным этическим комитетом ФГБУ «НМИЦ эндокринологии» Минздрава России (протокол № 17 от 28.10.2020).

## РЕЗУЛЬТАТЫ

Для пациентов с гиперандрогенией был характерен молодой возраст — 26 [ 23; 27] лет. Так как «физиологическая» гиперандрогения у мужчин клинически проявляется в основном кожными симптомами, все пациенты предъявляли жалобы либо на наличие акне, либо на алопецию, в ряде случаев эти симптомы сочетались. У всех обследованных пациентов уровни гликемии натощак, ренина, креатинина, СКФ, ионов крови, эстрадиола, а также показателей мультистероидного анализа крови соответствовали РИ, за исключением 17-гидроксипрогестерона, который у 2 пациентов с гиперандрогенией, обусловленной повышением ДГТ, и у 13 мужчин с гиперандрогенией, обусловленной повышением общего тестостерона, превышал верхнюю границу РИ. У 7 пациентов (4 из группы повышенного ДГТ и 3 из группы повышенного тестостерона) отмечалась гиперурикемия. У 11 мужчин (6 из группы повышенного ДГТ и 5 из группы повышенного тестостерона) была выявлена дислипидемия. Никто из пациентов жалоб на повышение АД не предъявлял. При измерении АД на приеме при осмотре пациента врачом также не было выявлено случаев его повышения. Данные обследования двух групп пациентов в сравнении представлены в таблице 1.

Были выявлены статистически значимые различия в уровнях 17-гидроксипрегненолона, 17-гидроксипрогестерона и андростендиона, которые были выше у мужчин с повышенным уровнем общего тестостерона. Статистически значимых различий в других показателях выявлено не было.

Случаев повышения АД в группе мужчин с повышением ДГТ выявлено не было. В группе мужчин с повышением уровня общего тестостерона было выявлено 8 пациентов с артериальной гипертензией (23,5%), различия между группами статистически значимы — p<0,001, точный критерий Фишера. Все случаи артериальной гипертензии были выявлены при анализе дневников давления, заполняемых пациентами, и подтверждены СМАД. Данные обследования пациентов из группы гиперандрогении, обусловленной повышением уровня общего тестостерона, в зависимости от наличия артериальной гипертензии представлены в таблице 2.

Были выявлены статистически значимые различия в уровне эстрадиола, который был ниже в группе пациентов с артериальной гипертензией, и показателях утреннего САД и ДАД. Другие исследуемые параметры статистически значимо не отличались.

Среднесуточные показатели АД, характерные для пациентов с артериальной гипертензией, представлены в таблице 3.

Для 6 пациентов из лиц с артериальной гипертензией по данным СМАД был характерен утренний подъем АД, а для 2 мужчин — вечерний, рисунок 1.

Интегративные показатели СМАД у пациентов с артериальной гипертензией представлены на рисунках 2 и 3.

Ни у одного из пациентов с артериальной гипертензией не отмечалось гипергликемии, патологических изменений креатинина, СКФ, ренина, калия, натрия, хлорида крови, альдостерона, отсутствовали нарушения сердечного ритма, альбуминурия, патологические изменения на ЭКГ, а также признаки изменений сосудов глазного дна. У 2 пациентов отмечался избыток массы тела (ИМТ 24,2 и 29,2 кг/м2), который у последнего из них сопровождался дислипидемией (ХС 5,9 ммоль/л, ЛПНП 3,9 ммоль/л, ЛПВП 1,4 ммоль/л, ТГ 1,3 ммоль/л) и гиперурикемией (мочевая кислота 735 мкмоль/л). Еще у одного пациента с нормальной массой тела также отмечалась дислипидемия (ХС 5,3 ммоль/л, ЛПНП 3,6 ммоль/л, ЛПВП 1,0 ммоль/л, ТГ 1,4 ммоль/л). Таким образом, диагноз у 7 пациентов соответствовал Гипертонической болезни 1 стадии. Степень АГ 1. Умеренный риск (Риск 2). И у одного пациента — Гипертонической болезни 1 стадии. Степень АГ 1. Умеренно-высокий риск (Риск 2-3).

Всем мужчинам с артериальной гипертензией были даны рекомендации по изменению образа жизни на 3 мес., однако выполнение этих рекомендаций привело к устранению артериальной гипертензии только у двух пациентов – пациента с избытком массы тела, гиперурикемией (также была устранена) и дислипидемией (также была устранена), и еще одного пациента с нормальной массой тела без дислипидемии. Еще у одного пациента была устранена дислипидемия, но сохранялась артериальная гипертензия. Следует отметить, что артериальная гипертензия была устранена только у пациентов с вечерним подъемом АД. Коррекции гиперандрогении не проводилось ни у одного из пациентов, тем не менее артериальная гипертензия была устранена у двух пациентов с вечерним подъемом АД, но ни у одного из пациентов с утренним подъемом АД. Таким образом, не было оснований считать артериальную гипертензию у пациентов с вечерним подъемом АД ассоциированной с гиперандрогенией, обусловленной повышением общего тестостерона. Интегративные данные СМАД пациентов (n=6) с артериальной гипертензией, ассоциированной с тестостероновой гиперандрогенией представлены на рисунках 4–6.

Даже после исключения двух пациентов с вечерним подъемом АД из сравнительного анализа, распространенность артериальной гипертензии, ассоциированной с гиперандрогенией, обусловленной повышением общего тестостерона составила 17,6%, что по-прежнему являлось статистически значимо выше таковой в группе с гиперандрогенией, обусловленной повышением ДГТ, p=0,001 (точный критерий Фишера). Сохранилась и статистическая значимость различий в уровнях эстрадиола, которые как и ранее были статистически значимо ниже у пациентов с артериальной гипертензией в сравнении с мужчинами с повышенным тестостероном, но без гипертензии (40,3 [ 29,3; 62,1] против 96,7 [ 83,9; 106,6] пмоль/л, p<0,001, U-критерий Манна–Уитни).

**Table:** Таблица 1. Результаты обследования пациентов в зависимости от типа гиперандрогении Примечание: Me [ 25%; 75%], *U-критерий Манна–Уитни. Так как проведены множественные сравнения, применена поправка Бонферрони, уровень статистической значимости p<0,001.

Параметр	Гиперандрогения ДГТ(n=66)	Гиперандрогения Тестостерон (n=34)	p
ДГТ, пг/мл	2472 [ 1995; 2888]	757 [ 645; 862]	<0,001
Общий тестостерон, нмоль/л	17,5 [ 16,0; 20,7]	36,3 [ 35,1; 38,2]	<0,001
Возраст, лет	25 [ 23; 27]	26 [ 24; 28]	0,187
ИМТ, кг/м2	24,6 [ 23,5; 26,4]	24,2 [ 22,9; 25,4]	0,489
ОТ, см	92 [ 86; 95]	90 [ 87; 96]	0,904
Глюкоза, ммоль/л	5,0 [ 4,6; 5,3]	5,2 [ 4,8; 5,3]	0,238
Мочевая кислота, мкмоль/л	370 [ 340; 400]	379 [ 354; 410]	0,135
ЛГ, ЕД/мл	4,0 [ 3,1; 4,9]	4,0 [ 3,3; 4,9]	0,922
ГСПГ, нмоль/л	30,0 [ 25,2; 38,1]	32,6 [ 26,0; 43,1]	0,287
Эстрадиол, пмоль/л	87,1 [ 71,6; 95,7]	93,9 [ 73,6; 105,2]	0,062
Кортизол, нмоль/л	335 [ 260; 419]	311 [ 259; 365]	0,187
Альдостерон, пмоль/л	168 [ 110; 278]	219 [ 116; 315]	0,145
Кортизон нмоль/л	53,6 [ 45,9; 61,2]	50,3 [ 43,2; 58,9]	0,164
21-деоксикортизол, нмоль/л	0,04 [ 0,01; 0,10]	0,02 [ 0,01; 0,05]	0,278
11-деоксикортизол, нмоль/л	0,9 [ 0,4; 1,4]	1,0 [ 0,9; 1,3]	0,061
17-гидроксипрегненолон, нмоль/л	2,12 [ 1,50; 2,80]	4,60 [ 3,67; 6,33]	<0,001
17-гидроксипрогестерон, нмоль/л	2,57 [ 1,68; 6,20]	5,83 [ 3,87; 9,83]	<0,001
Кортикостерон, нмоль/л	6,09 [ 3,00; 10,30]	6,86 [ 2,78; 10,30]	0,985
Деоксикортикостерон, нмоль/л	0,07 [ 0,03; 0,11]	0,09 [ 0,04; 0,16]	0,159
Прогестерон, нмоль/л	0,10 [ 0,10; 0,16]	0,17 [ 0,10; 0,20]	0,014
Прегненолон, нмоль/л	2,03 [ 1,39; 2,90]	2,37 [ 1,70; 3,17]	0,187
Андростендион, нмоль/л	3,05 [ 2,03; 4,00]	4,18 [ 3,42; 4,87]	<0,001
Дегидроэпиандростерон, нмоль/л	6,3 [ 3,4; 10,5]	10,4 [ 6,2; 11,9]	0,057
Ренин, мЕд/л	19,2 [ 14,4; 28,8]	26,1 [ 19,1; 35,6]	0,022
Креатинин, мкмоль/л	77,4 [ 71,4; 87,0]	81,0 [ 75,6; 89,4]	0,200
К, ммоль/л	4,4 [ 4,2; 4,7]	4,4 [ 4,2; 4,7]	0,991
Na, ммоль/л	141,0 [ 138,9; 142,8]	139,2 [ 138,2; 141,3]	0,011
Cl, ммоль/л	104,0 [ 102,0; 105,7]	103,0 [ 101,8; 105,2]	0,325
СКФ, мл/мин/1,73 м2	119 [ 110; 123]	115 [ 104; 120]	0,090
ХС, ммоль/л	4,4 [ 4,0; 4,7]	4,1 [ 4,0; 4,5]	0,244
ЛПНП, ммоль/л	2,2 [ 1,8; 2,7]	2,2 [ 1,8; 2,8]	0,698
ЛПВП, ммоль/л	1,7 [ 1,4; 2,0]	1,4 [ 1,2; 2,0]	0,394
ТГ, ммоль/л	1,0 [ 0,9; 1,3]	1,0 [ 0,9; 1,4]	0,241

**Table:** Таблица 2. Результаты обследования пациентов в зависимости от наличия артериальной гипертензии Примечание: Me [ 25%; 75%], *U-критерий Манна–Уитни. Так как проведены множественные сравнения, применена поправка Бонферрони, уровень статистической значимости p<0,001.

Параметр	Артериальная гипертензия(n=8)	Гипертензии нет(n=26)	p
Возраст, лет	26 [ 23; 28]	26 [ 24; 28]	0,705
ИМТ, кг/м2	23,9 [ 23,0; 25,2]	24,3 [ 22,9; 25,4]	0,795
ОТ, см	89 [ 86; 94]	91 [ 87; 96]	0,735
Глюкоза, ммоль/л	5,1 [ 4,7; 5,3]	5,2 [ 4,8; 5,3]	0,952
Мочевая кислота, мкмоль/л	379 [ 339; 412]	381 [ 355; 410]	0,705
ЛГ, ЕД/мл	4,0 [ 3,1; 4,5]	3,9 [ 3,3; 4,9]	0,675
ГСПГ, нмоль/л	36,0 [ 30,9; 46,3]	31,5 [ 24,9; 43,1]	0,288
ДГТ, пг/мл	820 [ 771; 848]	735 [ 597; 879]	0,252
Эстрадиол, пмоль/л	53,3 [ 32,7; 73,9]	98,2 [ 84,8; 107,4]	<0,001
Общий тестостерон, нмоль/л	36,0 [ 35,2; 39,0]	36,7 [ 35,1; 38,2]	0,675
Кортизол, нмоль/л	324 [ 275; 402]	300 [ 259; 352]	0,326
Альдостерон, пмоль/л	224 [ 140; 262]	216 [ 116; 344]	0,705
Кортизон, нмоль/л	52,5 [ 47,1; 61,9]	47,6 [ 39,8; 57,2]	0,288
21-деоксикортизол, нмоль/л	0,03 [ 0,02; 0,06]	0,02 [ 0,01; 0,04]	0,563
11-деоксикортизол, нмоль/л	0,9 [ 0,8; 1,1]	1,0 [ 1,0; 1,4]	0,270
17-гидроксипрегненолон, нмоль/л	4,7 [ 3,8; 6,5]	4,6 [ 3,6; 6,2]	0,705
17-гидроксипрогестерон, нмоль/л	7,06 [ 5,07; 11,45]	5,55 [ 3,83; 9,67]	0,326
Кортикостерон, нмоль/л	9,1 [ 6,1; 10,3]	5,9 [ 2,1; 9,9]	0,236
Деоксикортикостерон, нмоль/л	0,04 [ 0,02; 0,15]	0,10 [ 0,07; 0,16]	0,119
Прогестерон, нмоль/л	0,2 [ 0,1; 0,2]	0,1 [0,1; 0,2]	0,177
Прегненолон, нмоль/л	2,5 [ 1,7; 3,0]	2,3 [ 1,7; 3,4]	0,920
Андростендион, нмоль/л	3,86 [ 3,43; 4,90]	4,21 [ 3,42; 4,87]	0,984
Дегидроэпиандростерон, нмоль/л	11,6 [ 8,6; 14,2]	9,8 [ 5,9; 11,4]	0,129
Ренин, мЕд/л	24,2 [ 20,3; 41,8]	26,6 [ 17,2; 33,2]	0,765
Креатинин, мкмоль/л	86,4 [ 79,4; 93,1]	78,8 [ 73,0; 87,6]	0,190
К, ммоль/л	4,2 [ 4,1; 4,5]	4,5 [ 4,3; 4,7]	0,129
Na, ммоль/л	138,9 [ 138,4; 139,8]	139,3 [ 138,2; 142,0]	0,412
Cl, ммоль/л	102,5 [ 101,8; 103,7]	103,5 [ 101,8; 105,5]	0,510
СКФ, мл/мин/1,73м2	109 [ 98; 113]	117 [ 106; 122]	0,152
ХС, ммоль/л	4,4 [ 4,0; 5,1]	4,1 [ 4,0; 4,5]	0,460
ЛПНП, ммоль/л	2,2 [ 1,6; 3,2]	2,2 [ 1,9; 2,7]	0,705
ЛПВП, ммоль/л	1,4 [ 1,1; 1,4]	1,7 [ 1,2; 2,1]	0,252
ТГ, ммоль/л	0,8 [ 0,6; 1,3]	1,1 [ 1,0; 1,4]	0,220
САД утро, мм рт. ст.	141 [ 135; 142]	118 [ 116; 122]	<0,001
ДАД утро, мм рт. ст.	92 [ 87; 93]	80 [ 77; 82]	<0,001
САД день, мм рт. ст.	127 [ 125; 128]	126 [ 124; 128]	0,270
ДАД день, мм рт. ст.	84 [ 82; 86]	82 [ 81; 84]	0,052
САД вечер, мм рт. ст.	125 [ 123; 126]	126 [ 123; 127]	0,735
ДАД вечер, мм рт. ст.	83 [ 81; 88]	80 [ 79; 82]	0,004
САД перед сном, мм рт. ст.	124 [ 122; 126]	123 [ 120; 125]	0,119
ДАД перед сном, мм рт. ст.	80 [ 78; 82]	79 [ 78; 80]	0,057

**Table:** Таблица 3. Среднесуточные показатели артериального давления у пациентов с артериальной гипертензией (n=8), мм рт. ст.

Тип АД и номер пациента	Me	Min	Max	25%-квартиль	75%-квартиль
САД1	129	120	153	125	135
САД2	126	115	150	122	131
САД3	128	118	149	125	135
САД4	127	116	152	124	135
САД5	126	116	152	122	131
САД6	125	115	153	121	133
САД7	127	115	144	121	131
САД8	125	112	146	119	130
ДАД1	83	76	98	81	87
ДАД2	81	74	98	79	85
ДАД3	83	76	97	81	86
ДАД4	84	78	98	81	87
ДАД5	82	78	98	81	87
ДАД6	81	77	98	79	86
ДАД7	82	76	90	79	83
ДАД8	80	73	95	78	82

**Figure:**
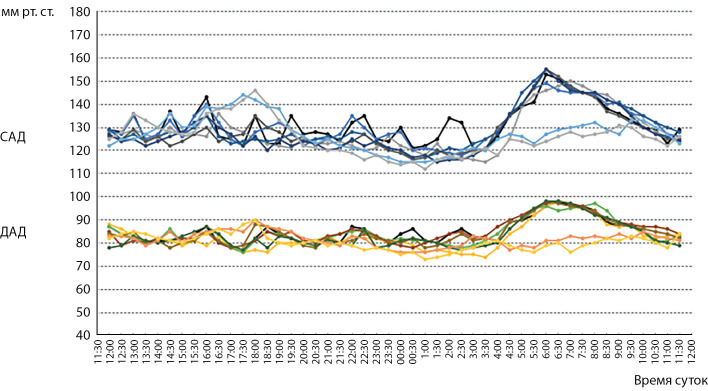
Рисунок 1. Показатели СМАД у пациентов с артериальной гипертензией (n=8).

**Figure:**
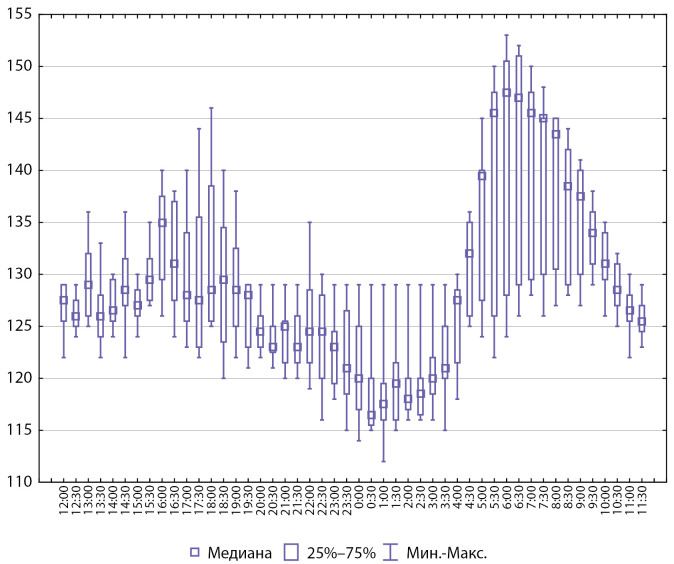
Рисунок 2. Интегративные показатели СМАД, систолическое АД (n=8).

**Figure:**
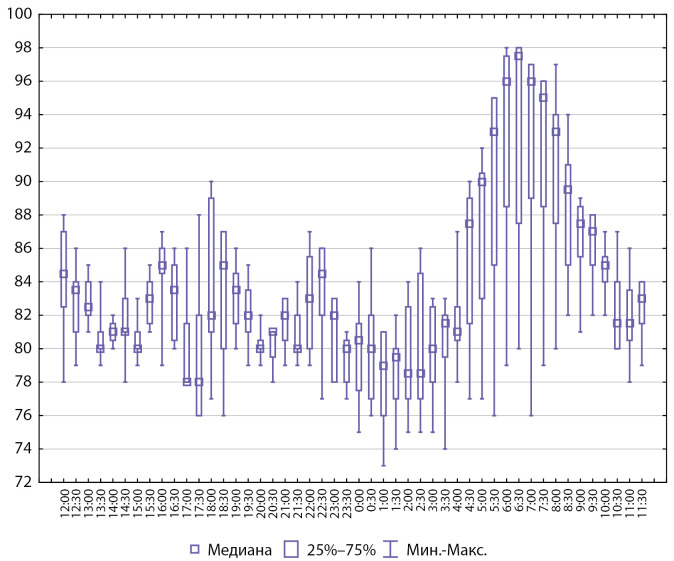
Рисунок 3. Интегративные показатели СМАД, диастолическое АД (n=8).

**Figure:**
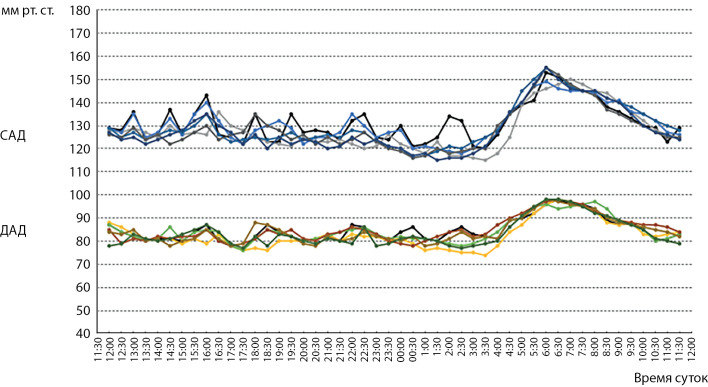
Рисунок 4. Показатели СМАД у пациентов с артериальной гипертензией (n=6).

**Figure:**
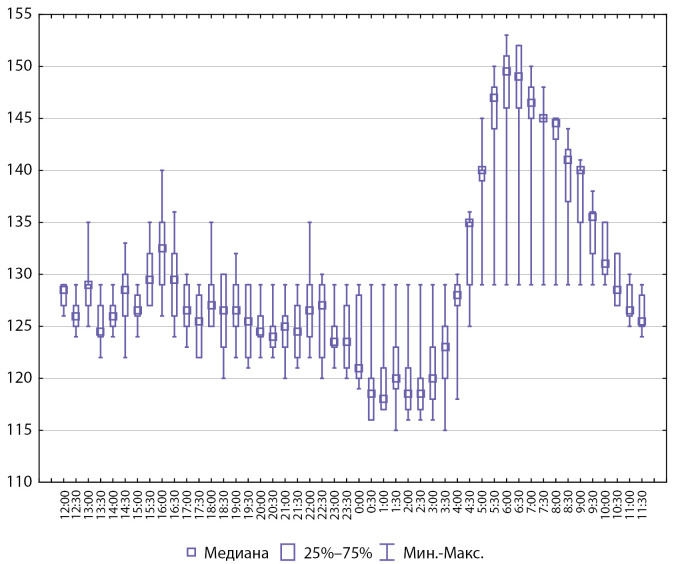
Рисунок 5. Интегративные показатели СМАД, систолическое АД (n=6).

**Figure:**
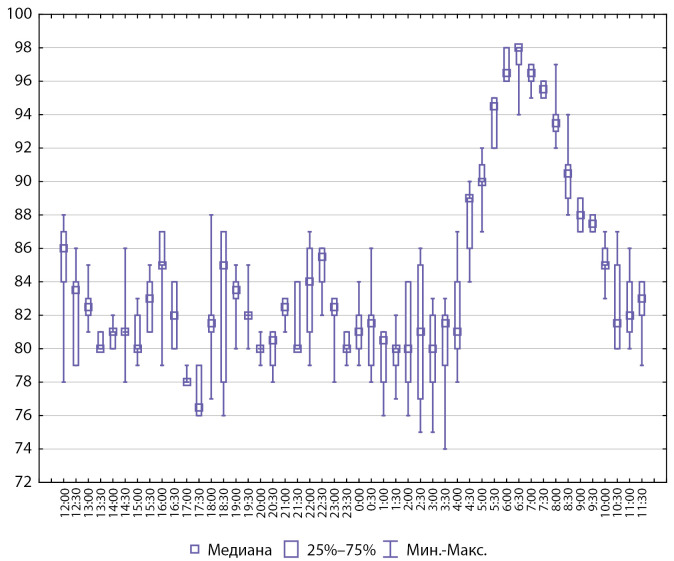
Рисунок 6. Интегративные показатели СМАД, диастолическое АД (n=6).

## ОБСУЖДЕНИЕ

Репрезентативность выборок

Учитывая, что пациентов в ФГБУ НМИЦ Эндокринологии Минздрава России направляли врачи из разных, не связанных между собой медицинских центров, из различных районов Москвы и Московской области, предполагается, что выборка репрезентативна для общей популяции мужчин с гиперандрогенией. Однако, все же возможно искажение репрезентативности в отношении российской популяции по уровню образования, образу жизни и доходов пациентов.

Сопоставление с другими публикациями

Вопрос гиперандрогении у мужчин рассматривался рядом исследователей, как мужской аналог СПКЯ [11][12]. Так, в 2021 году на конгрессе ENDO 2021 была представлена работа, в которой Jia Zhu и соавт. предложили алгоритм прогнозирования рисков аналога СПКЯ у мужчин и его осложнений. Алгоритм был создан на основе крупномасштабного метаанализа пациенток с СПКЯ, где результаты были адаптированы для выявления ассоциаций между кардио-метаболическими и андрогенными фенотипами у мужчин. При этом были выявлены ассоциации между метаболическими рисками, рисками сердечно-сосудистых заболеваний и уровнем тестостерона, что было рассмотрено как мужской аналог СПКЯ [13]. Согласно результатам нашего исследования, у мужчин с «физиологической» гиперандрогенией встречались такие метаболические нарушения, как гиперурикемия и дислипидемия, которые, однако, были малочисленны и легко скорректированы путем модификации образа жизни, что позволяет предполагать их патогенез, не связанный с повышенными уровнями андрогенов.

По результатам проведенного исследования можно выделить несколько подгрупп пациентов с «физиологической» гиперандрогенией — пациенты с повышенным уровнем общего тестостерона, пациенты с гиперандрогенией, обусловленной изолированным повышением дигидротестостерона (ДГТ), а также их сочетанием. Повышения АД в группе пациентов с изолированно повышенным уровнем ДГТ не встречалось, что может быть обусловлено тем, что ДГТ принимает участие в регуляции сосудистого сопротивления и артериального давления лишь за счет вазорелаксации, опосредованной блокирующим влиянием на L-VOCC каналы (потенциал-управляемые кальциевые каналы L-типа, которые принимают участие в регуляции сокращения артериальных сосудов), оказывая скорее антигипертензивное действие [14]. В группе пациентов с «физиологической» гиперандрогенией, обусловленной повышением уровня общего тестостерона и артериальной гипертензией, наблюдалось патологическое повышение САД и ДАД в утренние часы, которое может быть обусловлено пиками выработки тестостерона [15]. Это требует повышенного внимания, так как установлено, что утренняя гипертензия связана с поражением органов-мишеней и неблагоприятными сердечно-сосудистыми исходами [16][17].

В группе пациентов с «физиологической» гиперандрогенией, обусловленной повышением уровня общего тестостерона установлены следующие особенности стероидогенеза: статистически значимое повышение уровней андростендиона, 17-гидроксипрегненолона и 17-гидроксипрогестерона, который является сывороточным маркером уровня продукции интратестикулярного тестостерона [18]. Повышение этих показателей позволяет предположить повышенную активность ферментов 3-бета-гидроксистероиддегидрогеназы и 17а-гидроксилазы/17,20-лиазы (Р415с17), что, возможно, и обуславливает повышение продукции андрогенов, но не сопровождается нарушением синтеза кортизола и альдостерона. Кроме того, для пациентов с артериальной гипертензией был характерен статистически значимо более низкий уровень эстрадиола (при этом находящийся в пределах РИ), чем в группе пациентов без артериальной гипертензии, что согласуется с концепцией протективного действия эстрогенов на АД [19][20]. Современные исследования предполагают, что эстрадиол обуславливает свои вазопротективные свойства путем усиления ангиогенеза и вазодилатации, а также снижения свободных радикалов кислорода в эндотелии, окислительного стресса и фиброза [20][21]. В этих работах также указывается, что эстрадиол благодаря вышеописанным механизмам ограничивает ремоделирование сердца и препятствует развитию его гипертрофии.

Клиническая значимость результатов

Наличие бессимптомной артериальной гипертензии у мужчин с гиперандрогенией, обусловленной повышением общего тестостерона, диктует необходимость регулярного самоконтроля АД, несмотря на молодой возраст. Ассоциация артериальной гипертензии с повышенными уровнями тестостерона требует внимания у пациентов, получающих лечение препаратами тестостерона, дающими супрафизиологические пики его концентрации.

Ограничения исследования

Ограничениями исследования являются возможные проблемы с репрезентативностью выборки в отношении общей популяции (указаны в соответствующем разделе).

Направления дальнейших исследований

В продолжении проведенного исследования планируется изучить методы коррекции артериальной гипертензии у мужчин с гиперандрогений.

## ЗАКЛЮЧЕНИЕ

При «физиологической» гиперандрогении, обусловленной повышением уровня ДГТ, случаев артериальной гипертензии не наблюдается, в то время как ее частота встречаемости при «физиологической» гиперандрогении, обусловленной повышением общего тестостерона, составила 8%. В 6% случаев ее можно считать ассоциированной именно с тестостероновой гиперандрогенией. Для гипертензии были характерны бессимптомное течение, повышение АД в ранние утренние часы, степень артериальной гипертензии 1, умеренный риск. Особенностями стероидогенеза являлись повышенная выработка 17-гидроксипрегненолона, 17-гидроксипрогестерона и андростендиона у мужчин с гиперандрогенией, обусловленной повышением общего тестостерона, и сниженная выработка эстрадиола у пациентов с артериальной гипертензией в сравнении с пациентами без таковой при тестостероновой гиперандрогенией.

## ДОПОЛНИТЕЛЬНАЯ ИНФОРМАЦИЯ

Источник финансирования. Исследование и публикация статьи осуществлены на личные средства авторского коллектива.

Конфликт интересов. Авторы данной статьи подтвердили отсутствие конфликта интересов, о котором необходимо сообщить.

Выражение признательности. Авторы выражают искреннюю благодарность пациентам, принявшим участие в проведении исследования.

Участие авторов. Филатова В.А. — существенный вклад в получение, анализ данных и интерпретацию результатов; написание статьи; Роживанов Р.В. — существенный вклад в концепцию и дизайн исследования, анализ данных и интерпретацию результатов; Бондаренко И.З. — существенный вклад в анализ данных и интерпретацию результатов; Иоутси В.А. — существенный вклад в получение данных лабораторного обследования; Андреева Е.Н. — существенный вклад во внесение правки в рукопись с целью повышения научной ценности статьи; Мельниченко Г. А. — существенный вклад во внесение правки в рукопись с целью повышения научной ценности статьи; Мокрышева Н.Г. — существенный вклад во внесение правки в рукопись с целью повышения научной ценности статьи. Все авторы одобрили финальную версию статьи перед публикацией, выразили согласие нести ответственность за все аспекты работы, подразумевающую надлежащее изучение и решение вопросов, связанных с точностью или добросовестностью любой части работы.
